# Graduated Controlled Atmosphere: A Novel Approach to Increase “Duke” Blueberry Storage Life

**DOI:** 10.3389/fpls.2020.00221

**Published:** 2020-03-17

**Authors:** Natalia Falagán, Tiana Miclo, Leon A. Terry

**Affiliations:** Plant Science Laboratory, Cranfield University, Cranfield, United Kingdom

**Keywords:** *Vaccinium corymbosum* L, postharvest, quality, cold storage, controlled atmosphere

## Abstract

Blueberries (*Vaccinium corymbosum* L.) are highly valued for their health-promoting potential, yet they are extremely perishable. Controlled atmosphere (CA) strategies reduce blueberry respiratory metabolism, slowing down senescence. However, the sudden change of atmosphere could elicit a physical abiotic stress in the fruit, negatively affecting quality. We propose an innovative approach based on controlled graduation to slowly reach optimum gas storage conditions as an alternative to standard CA. For two consecutive seasons, “Duke” blueberries were subjected to four different storage conditions: control (air); standard CA (sudden exposure to 5 kPa O_2_ and 10 kPa CO_2_ across the experiment); GCA3 and GCA7 (gradually reaching 5 kPa O_2_ and 10 kPa CO_2_ in 3 and 7 days, respectively). Fruit were stored for 28 days at 0 ± 0.5°C. Real-time respirometry provided an in-depth insight to the respiratory response of blueberries to their gas environment. Blueberries subjected to the graduated application of CA (GCA) treatments had a lower steady-state respiration rate compared to control and standard CA fruit. This indicated a reduction in metabolic activity that positively impacted quality and storage life extension. For example, GCA3 and GCA7 blueberries had a 25% longer storage life when compared to control, based on reduced decay incidence. In addition, GCA fruit were 27% firmer than control and CA fruit after 28 days of cold storage. GCA3 had a positive effect on maintaining individual sugars concentrations throughout the experiment, and both GCA treatments maintained ascorbic acid content close to initial values compared to a decrease of 44% in the control fruit at the end of the experiment. This work provides a paradigm shift in how CA could be applied and a better understanding of blueberry physiology and postharvest behavior.

## Introduction

Blueberries (*Vaccinium* spp.) have become a popular soft fruit because of their organoleptic characteristics and health-promoting compound content (Manganaris et al., 2014). Yet, blueberries are highly perishable. Their storage life at 0°C varies between 14 and 20 days depending on preharvest factors (i.e., cultivar, ripeness stage, harvest method) and storage conditions (Matiacevich et al., 2013). Slowing down respiration, the main process in fruit metabolism (Gomes et al., 2010), is key to delaying senescence. Respiration is mostly affected by temperature and the respiratory gaseous environment (Wang et al., 2019). Hence, the combination of low temperature storage and controlled atmosphere (CA) technology has been used for many years to maintain physical and functional quality (Terry et al., 2009). For blueberries, a high concentration of carbon dioxide (CO_2_) has a positive impact on decay suppression (Retamales and Hancock, 2018). Recommendations range between 10–12 kPa CO_2_; higher levels elicit a negative effect on firmness, flavor, and titratable acidity (TA) content (Kader, 2003; Harb and Streif, 2004). Oxygen (O_2_) concentration has less impact on blueberry quality, although lowering it to 2–5 kPa is advised (Kader, 2003). O_2_ concentrations below 2 kPa lead to hypoxia and fermentation (Retamales and Hancock, 2018). However, applying standard CA can lead to abiotic stress derived from a sudden change in the surrounding atmosphere, negatively affecting quality (Falagán and Terry, 2018). The introduction of real-time respirometry has allowed the effects of CA to be monitored, reactivating research in this field. Previous studies have shown how targeted CA applications had a similar effect to continuous CA in extending shelf-life of onion, strawberry and avocado (Chope et al., 2007; Alamar et al., 2017). Based on these results, we hypothesized in this work that the graduated application of CA (GCA) could avoid this metabolic shock as it allows fresh produce to more slowly adapt to an optimal gaseous environment. This approach is based on the way modified atmosphere packaging (MAP) works, gradually reaching the respiratory equilibrium, instead of exposing the fresh produce to a sudden change in the atmosphere. Work on adaptive control of the gas diffusion in MAP has been successfully implemented before, e.g., preserving quality in blueberries and spinach (Lee et al., 2016). In this study, the MAP approach is translated to CA systems. The aim of this work was to study the benefits of GCA on the physiological and functional quality of “Duke” blueberries.

## Materials and Methods

### Plant Material

Blueberries (*Vaccinium corymbosum* L.) “Duke” were hand harvested at Cobrey Farms (Herefordshire, United Kingdom) on the 12 of July 2017 (season 1) and on the 27 of June 2018 (season 2) at their optimal maturity stage according to commercial standards defined by the company, considering color and maturity index. They were immediately transported in 600 g punnets to the Plant Science Laboratory at Cranfield University by refrigerated truck (2.5 h). Fruit were then directly transferred to the treatment boxes upon arrival at the laboratory and allowed to cool down for 24 h at 0°C prior treatment application.

### Experimental Plan

**FIGURE 1:**
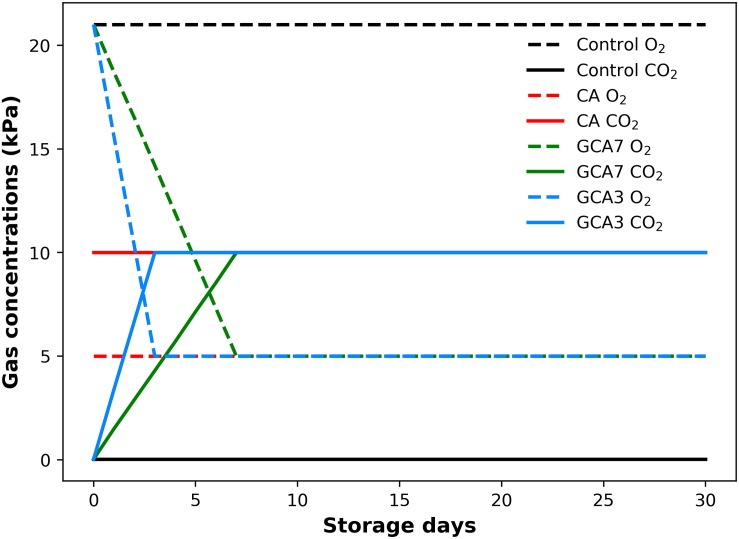
Gas concentration (CO_2_ and O_2_; kPa) supply throughout 28 days of storage at 0°C and 90–95 RH%; air [control]; standard CA (10 kPa CO_2_ + 5 kPa O_2_); graduated CA 7 (GCA7; 7 days to achieve standard CA values); and GCA3 (3 days to achieve standard CA values).

Forty-eight 4 L air-tight polypropylene boxes (L&L Nordic OÜ, Estonia) were used to store 1 kg of sound fruit each. They were kept in a cold room at 0°C and 90–95% relative humidity (RH). Four treatments were considered: (i) Control, flushed with regular atmosphere gas concentrations (20.9 kPa O_2_ and 0.03 kPa CO_2_); (ii) standard CA (5 kPa O_2_ and 10 kPa CO_2_); (iii) graduated CA 3 (GCA3) flushed to reach the desired CA partial pressures in 3 days; and (iv) GCA7 gradually flushed to reach the standard CA conditions in 7 days (Figure 1). Each condition was performed in triplicate. The combination of 5 kPa O_2_ and 10 kPa CO_2_ was selected as optimal according to previous work (Kader, 2003; Harb and Streif, 2004; Retamales and Hancock, 2018). For each CA condition, boxes were connected to an ICA6000 (International Controlled Atmosphere Ltd., Paddock Wood, Kent, United Kingdom) via PVC tubes; gases were bubbled through water to maintain a high RH%. For each treatment, an extra empty box was used as a baseline to avoid cross-contamination and allow respirometry calculations. The ICA6000 included an automated sample sequencing system to measure and control the gas concentration introduced in CA environments. The system was regularly checked for calibration with both fresh air and bottled calibration gas. Both temperature and RH were monitored in real-time with RD Sens RFS-TH (Prodisei, Valencia, Spain). Sampling was carried out on days 0, 7, 14, 21, and 28. Day 0 (one day after harvest) analysis were considered as baseline and samples were taken before placing the fruit in the treatment boxes.

### Fruit Quality Assessment

#### Respiration Rate

Real-time respiration rate was continuously recorded using a Sable Respirometry System (model 1.3.8 Pro, Sable Systems International, Nevada, United States) as described in Alamar et al. (2017) with slight modifications. Air from each box passed through a CA-10 (Firmware version 1.05) and a FC-10 sensor (Firmware version 3.0), which recorded CO_2_ and O_2_ partial pressures (kPa), respectively. For each treatment, an empty box was used as a baseline. These baseline boxes were sampled for 2 min between each replicate and for 6 min between each treatment to avoid cross-contamination. Baseline values were subtracted from sample values to obtain an accurate respiration rate expressed in mg kg^–1^ h^–1^.

#### Decay and Weight Loss

Fruit presenting fungal development symptoms, leakage, or collapse were considered as decayed. Decay was assessed on 100 blueberries per replicate and results were expressed as percentages. Decay severity was evaluated using a scale, where 1 = 0%, 2 = 1 to 25%, 3 = 26 to 50%, 4 = 51 to 75%, and 5 = 76 to 100% of the decayed fruit. Fruit were counted and a percentage was attributed to each level of the scale. Weight was recorded weekly using a digital balance (Precisa Ltd., Buckinghamshire, United Kingdom) with 0.1 g resolution. The same boxes were measured throughout the experiment. Weight loss was expressed as the percentage of loss compared to the initial 1 kg weight (Paniagua et al., 2014).

#### Firmness

A non-destructive compression test was performed on ten fruit per replicate using a uniaxial texture analyzer (model 5542, Instron, Norwood, MA, United States) equipped with a 38 mm diameter flat steel disc and a calibrated 500 N load cell. Berries were compressed by 2 mm, equatorially, at a rate of 1.2 mm s^–1^ according to Schotsmans et al. (2007) with slight modifications. The peak force necessary to achieve the target distance was recorded. Firmness was expressed as Newton (N).

#### pH, Total Soluble Solids, and Titratable Acidity

The juice of 25 blueberries per replicate (Schotsmans et al., 2007) was extracted using a commercial blender (Moulinex, Berkshire, United Kingdom). pH was recorded using a pH-meter (model 3540, Jenway, Staffordshire, United Kingdom). Total soluble solids (TSS) were determined using a digital refractometer (model PR-32α, Atago Ltd., Tokyo, Japan). TA was determined by titrating a solution of 5 mL of juice diluted in 45 mL of distilled water to pH 8.4 using 0.1 N NaOH and an automatic titrator (Mettler Toledo Ltd., Leicestershire, United Kingdom), according to Zheng et al. (2008) with slight modifications. Results were expressed as g citric acid L^–1^ (Schotsmans et al., 2007).

### Determination of Biochemistry Attributes

#### Sugar Content

For each replicate (*n* = 3), following Downes and Terry (2010) with modifications, freeze-dried blueberry powder (150 mg) was extracted and then analyzed in an HPLC (Agilent Technologies 1200 series, Berkshire, United Kingdom) with an evaporative light scattering detector (ELSD, Agilent Technologies 1200 Series, G1362A). Sugars were quantified using the external standards glucose, fructose and sucrose purchased from Sigma-Aldrich.

#### Organic Acids Content

For each replicate (*n* = 3), following Crespo et al. (2010) with modifications, freeze-dried blueberry powder (50 mg) was extracted and analyzed in an HPLC (Agilent Technologies 1200 series, Berkshire, United Kingdom) equipped with a diode array detector (DAD Agilent Technologies 1200 Series, G1315B) set at 210 nm was used to quantify ascorbic and citric acids (Sigma Aldrich, Kent, United Kingdom). Separation was performed on a prevail organic acid column of 250 mm × 4.6 mm, 5 μm particle size (Hichrom, United Kingdom; Part no. 88645). Mobile phase was 25 mM KH_2_PO_4_ in water, adjusted to pH 2.5 using meta-phosphoric acid. Organics acids were quantified using the external standards ascorbic and citric acids purchased from Sigma-Aldrich.

### Statistical Analysis

Data were subjected to analysis of variance (ANOVA) using GenStat for Windows (8.1, VSN International Ltd., Hertfordshire, United Kingdom). ANOVA assumptions were tested and found to be valid for this dataset. The differences between treatments and storage times were studied. Also, the differences between the two seasons were analyzed. Least significant difference values (LSD; *p* < 0.05) were calculated from each analysis.

## Results

### Respiratory Response to GCA

**FIGURE 2:**
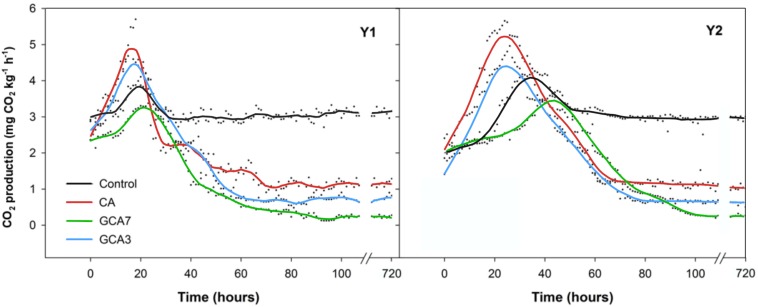
Real-time respiration rate (CO_2_ production expressed as mg kg^–1^ h^–1^) of “Duke” blueberry fruit subjected to controlled atmosphere (CA) applications throughout 28 days of storage at 0°C and 90–95 RH%; air [control]; standard CA (10 kPa CO_2_ + 5 kPa O_2_); graduated CA 7 (GCA7; 7 days to achieve standard CA values); and GCA3 (3 days to achieve standard CA values). Two consecutive seasons are considered 2017 (year 1; Y1) and 2018 (Y2). Lines represent smooth fit curves.

A peak in CO_2_ production was observed for all treatments in both seasons; its intensity and timing varied depending on treatment. Blueberries subjected to standard CA conditions showed the earliest and highest CO_2_ burst, while GCA7 was the most delayed and had the lowest peak. CO_2_ production reached a plateau after *ca.* 60 h in both years; control samples presented the highest CO_2_ production compared to any of the CA treatments during the plateau period (Figure 2).

### Physiological Response to GCA

**FIGURE 3:**
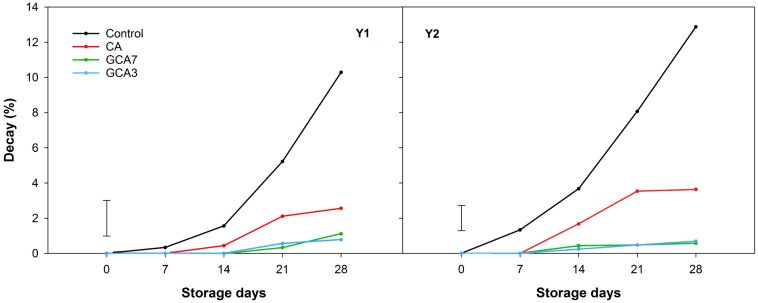
Decay expressed as percentage (%) of “Duke” blueberry fruit subjected to controlled atmosphere (CA) applications throughout 28 days of storage at 0°C and 90–95 RH%; air [control]; standard CA (10 kPa CO_2_ + 5 kPa O_2_); graduated CA 7 (GCA7; 7 days to achieve standard CA values); and GCA3 (3 days to achieve standard CA values). Two consecutive seasons are considered 2017 (year 1; Y1) and 2018 (Y2). Data represents means (*n* = 300). Least Significant Differences (LSD) are included as LSD_treatment*time_ = 1.0145 for Y1 and LSD_treatment*time_ = 0.713 for Y2.

**FIGURE 4:**
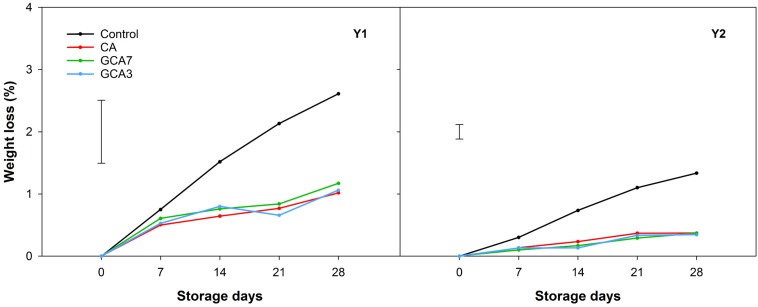
Weight loss expressed as percentage (%) of “Duke” blueberry fruit subjected to controlled atmosphere (CA) applications throughout 28 days of storage at 0°C and 90–95 RH%; air [control]; standard CA (10 kPa CO_2_ + 5 kPa O_2_); graduated CA 7 (GCA7; 7 days to achieve standard CA values); and GCA3 (3 days to achieve standard CA values). Two consecutive seasons are considered 2017 (year 1; Y1) and 2018 (Y2). Data represents means (*n* = 3). Least Significant Differences (LSD) are included as: LSD_treatment*time_ = 1.0145 for Y1 and LSD_treatment_ = LSD_time_ = 0.2338 for Y2.

GCA3, GCA7 and CA blueberries showed a significantly lower decay incidence than the control (92, 89, and 75% less than control, respectively; mean values between Y1 and Y2 values) after 28 storage days (Figure 3). In the case of decay severity, both GCA treatments prevented the development of fungal growth (<1%), while CA reached an average incidence value of 3.09% after 28 days (data not shown). Fruit subjected to CA and GCA showed less weight loss compared to the control. Both GCA delayed decay incidence by 14 days, compared to control and CA treatments (Figure 4). No significant differences were found between seasons (*p* < 0.05).

**FIGURE 5:**
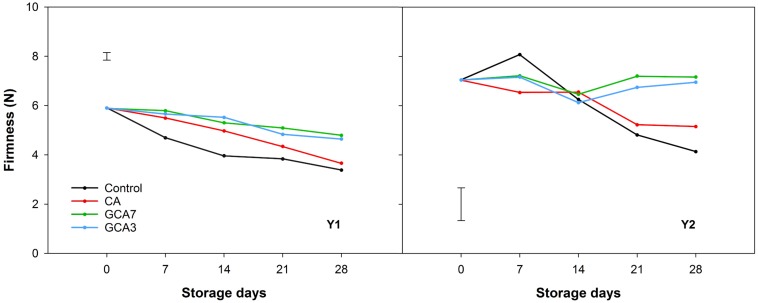
Firmness expressed as Newton (N) of “Duke” blueberry fruit subjected to controlled atmosphere (CA) applications throughout 28 days of storage at 0°C and 90–95 RH%; air [control]; standard CA (10 kPa CO_2_ + 5 kPa O_2_); graduated CA 7 (GCA7; 7 days to achieve standard CA values); and GCA3 (3 days to achieve standard CA values). Two consecutive seasons are considered 2017 (year 1; Y1) and 2018 (Y2). Data represents means (*n* = 30). Least Significant Differences (LSD) are included as: LSD_treatment_ = LSD_time_ = 0.310 for Y1 and LSD_treatment*time_ = 1.335 for Y2.

At the end of the storage period, control and CA blueberries lost an average of 40 and 29% of their initial firmness in Y1 and Y2, respectively. Firmness of berries held under GCA3 and GCA7 showed a loss of 16% in the first season and no significant changes were observed during the second season (Figure 5). pH showed a decreasing trend in Y1 and Y2, triggered by the interaction of treatment and time in Y1 and time in Y2. No significant differences were found in TA across both years (Supplementary Table 1).

### Biochemical Response to GCA

**FIGURE 6:**
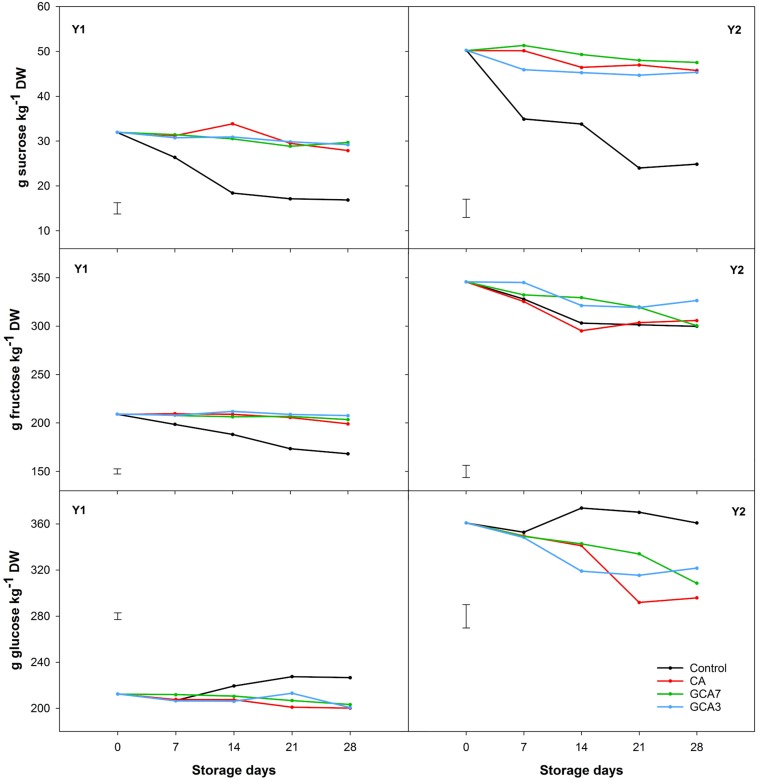
Individual sugar concentration (sucrose, fructose, and glucose) expressed as g kg^–1^ dry weight (DW) of “Duke” blueberry fruit subjected to controlled atmosphere (CA) applications throughout 28 days of storage at 0°C and 90–95 RH%; air [control]; standard CA (10 kPa CO_2_ + 5 kPa O_2_); graduated CA 7 (GCA7; 7 days to achieve standard CA values); and GCA3 (3 days to achieve standard CA values). Two consecutive seasons are considered 2017 (year 1; Y1) and 2018 (Y2). Data represents means (*n* = 3). Least Significant Differences (LSD) are included as: (i) Sucrose – LSD_treatment_ = LSD_time_ = 2.484 for Y1 and LSD_treatment_ = 4.082 for Y2; Fructose – LSD_treatment*day_ = 5.251 for Y1 and LSD_treatment_ = LSD_time_ = 12.63 for Y2; and Glucose – LSD_treatment*day_ = 5.848 for Y1 and LSD_treatment*time_ = 20.26 for Y2.

**FIGURE 7:**
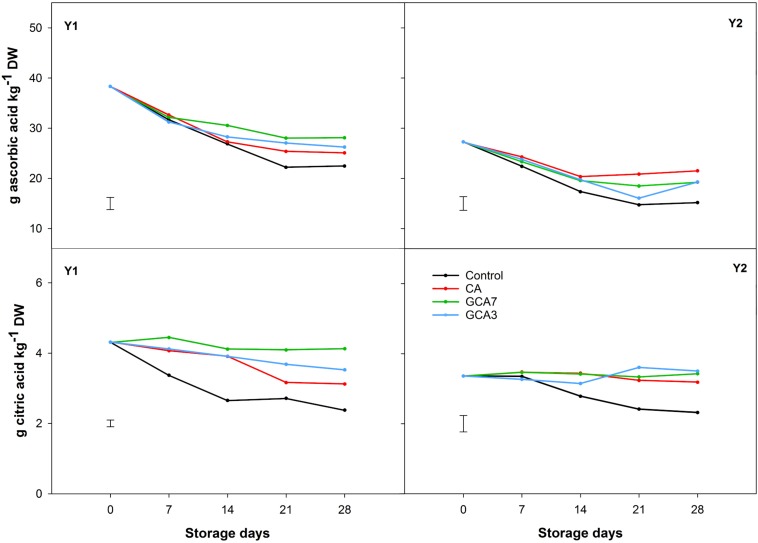
Organic acid concentration (ascorbic and citric acids) expressed as g kg^–1^ dry weight (DW) of “Duke” blueberry fruit subjected to controlled atmosphere (CA) applications throughout 28 days of storage at 0°C and 90–95 RH%; air [control]; standard CA (10 kPa CO_2_ + 5 kPa O_2_); graduated CA 7 (GCA7; 7 days to achieve standard CA values); and GCA3 (3 days to achieve standard CA values). Two consecutive seasons are considered 2017 (year 1; Y1) and 2018 (Y2). Data represents means (*n* = 3). Least Significant Differences (LSD) are included as: (i) ascorbic acid – LSD_treatment_ = LSD_time_ = 2.417 for Y1 and LSD_treatment_ = 2.731 for Y2; and citric acid – LSD_treatment_ = LSD_time_ = 0.1907 for Y1 and LSD_treatment_ = 0.4680 for Y2.

Total sugar content was 40% higher in Y2 compared to Y1, but showed a similar pattern during storage. CA, GCA3 and GCA7 maintained the concentration of sucrose and fructose across the experiment, while control blueberries presented a sharp decrease in sucrose (49% mean for Y1 and Y2) and fructose (12.5% mean for Y1 and Y2). Glucose concentrations were maintained in CA and GCA treated fruit, whilst they slightly increased in control fruit (Figure 6). Ascorbic acid content degraded by 34, 29, and 41% during storage for CA, GCA, and control blueberries, respectively, for Y1; and 21, 29, and 44% during storage for CA, GCA, and control blueberries, respectively, for Y2 (Figure 7). In the case of citric acid, no significant differences were found among CA treatments and time during the experiment. CA treatments maintained the initial citric acid level, while control samples suffered a decrease of 45 and 31% in Y1 and Y2, respectively (Figure 7). Significant differences were observed between Y1 and Y2 for both ascorbic and citric acids contents (Ascorbic acid: LSD_*Y*__1__×__*Y*__2_ = 1.56; citric acid: LSD_*Y*__1__×__*Y*__2_ = 0.23; *p* < 0.05). Despite these differences, the response of blueberries to CA and GCA was the same in both seasons (Figure 7).

## Discussion

Fruit and vegetables undergo physiological and functional changes during senescence, which affect their quality and storage life (Terry, 2011). Most of these changes are linked to fruit respiration, which supports the many metabolic reactions occurring in a cell (Saltveit, 2019). According to Van Hoorn (2004), the higher the respiration rate, the faster the fruit/vegetable metabolism, leading to early senescence. Until now, the main way to achieve lower blueberry respiration rate has been through cold storage and the modification of gas concentrations surrounding the fresh produce (lowering O_2_ availability and increasing CO_2_ concentration). This approach works because respiratory metabolism is affected by low O_2_ concentrations due to limited gas transport (Armstrong and Beckett, 2011), triggering the active regulation of respiration (Gupta et al., 2009). In this study we proposed a novel application of CA, based on reaching the optimal CA gas concentrations in a gradual way, preventing abiotic stress that results from the sudden change in the gaseous environment. The observed CO_2_ production bursts occurred at different times and were linked to the decrease of available O_2_ in the storage environment. Blueberries subjected to CA were exposed to a sudden drop in the available O_2_ from 21 to 5 kPa. In response to this change, CA samples presented a well-defined peak in CO_2_ production. Following CA response, GCA3 blueberries increased their CO_2_ production when the O_2_ availability dropped from 21 to 15.6 kPa. In the case of GCA7, its peak was delayed, coinciding with an O_2_ availability of *ca.* 15 kPa. Therefore, O_2_ concentrations below 15 kPa were seen to induce an abiotic stress, and consequently an increase in CO_2_ production. This finding agreed with Gupta et al. (2009) who highlighted the importance of the timescale in O_2_ availability changes for respiratory metabolism. These peaks in CO_2_ production represented an increase in respiration, interpreted as possible abiotic stress to the changing gaseous environment (Mathooko et al., 2001). Interestingly, control blueberries also showed a peak, although smaller than the treatments had provoked, before reaching a steady-state level. This phenomenon might be because of the handling procedure, which involved a change in temperature, RH and storage environment. More research is needed in this area to identify the mechanisms behind this response. Nevertheless, it is observed that a slower imposed GCA, such as GCA7, produced the most delayed and lowest peak in CO_2_ production compared to standard CA, GCA3 and control (Figure 2). We herein hypothesized that GCA allowed the adaptive response of respiration to low O_2_ availability, preventing the metabolic shock that could reduce the physical quality of the fruit (Armstrong and Beckett, 2011). Apart from preventing this abiotic stress, GCA achieved a lower respiration rate compared to standard CA and control treatments when it reached stability after *ca.* 7 days of storage.

In fresh produce, senescence is generally marked by changes in firmness, weight and decay incidence (Defraeye et al., 2015). The reduction in respiratory metabolism had a positive impact on firmness, which is a critical quality index for blueberry, as consumers associate it with freshness (Chiabrando and Giacalone, 2017). Changes in firmness are a consequence of primary cell wall component degradation, such as pectin, cellulose and hemicellulose (Liu et al., 2019). We speculated that low O_2_ and high CO_2_ storage environments inhibited the action of polygalacturonase and pectin methylesterase (Beaudry, 1999), which are the enzymes responsible for the catabolism of cell wall metabolism (Yang et al., 2018). This event was also observed in blueberries stored under standard CA conditions (Concha-Meyer et al., 2015). GCA regulated response and delayed firmness loss when compared to control and CA treatments. Previous studies showed that targeted CA application on strawberry maintained their color (flesh and calyx) and firmness when compared to control and standard CA (Alamar et al., 2017). Firmness loss is directly linked to weight loss and decay incidence, as observed in this study (Paniagua et al., 2014; Chen et al., 2017). In the case of weight loss, blueberries subjected to GCA showed the same response as CA fruit, both significantly lower than control samples. Weight is generally lost through transpiration, the gradient of water vapor pressure in the cell tissues (Díaz-Pérez et al., 2007). In blueberries, transpiration generally happens through the stem scar rather than through the cuticle and it increases with temperature (Moggia et al., 2017). The blueberries herein were stored at 0°C, so that overall low weight loss percentages were observed. Although control samples showed a significantly higher weight loss than CA and GCA blueberries, it is considered that losses up to 4–5% do not affect fruit freshness (Mannozzi et al., 2017). In this study, higher weight and firmness loss contributed to greater decay in control samples when compared to CA and GCA. In general, CA with CO_2_ concentrations over 6 kPa retard decay symptoms in blueberry fruit (Schotsmans et al., 2007; Rodríguez and Zoffoli, 2016). However, a long exposure to high CO_2_ levels can cause internal damage to fruit leading to firmness and weight loss. We assumed that a graduated CA approach prevented this damage, providing better quality fruit than CA. GCA is an innovative approach to standard CA storage, and is analogous to a MAP strategy. Alternatives to standard CA include targeted CA application, which showed that 2.5 days of CA were sufficient to extend the shelf-life of strawberry by 3 days based on delayed decay incidence (Alamar et al., 2017).

Sugars and organic acids are considered the main substrates in the respiratory metabolism (Saltveit, 2019). Recent studies have considered that glucose and sucrose can play a role as signaling molecules in the stress responses (Huang et al., 2016), rather than just as a carbon source. It has also been reported that in non-climacteric fruit such as strawberry both sucrose and glucose promote fruit ripening and senescence (Jia et al., 2013; Cherian et al., 2014). In this study, control samples showed sucrose hydrolysis (Figure 6), likely through the action of invertase and sucrose synthase (Thomas et al., 2016). This process was not observed in CA nor GCA, indicating an inhibition of sugar degradation and delayed senescence. The different sugar profiles between the control and CA/GCA blueberries was linked to observed respiratory behavior, confirming the role of sugars as substrates in the respiratory metabolism. However, there was no evidence to support the hypothesis of sugars acting as signaling molecules in the stress responses of blueberries, this being in agreement with Karppinen et al. (2018). In this study, there was a poor correlation between sugar content and TSS, as described in Terry (2012). With respect to TA, neither time or treatments had an effect on its content. In contrast, Duarte et al. (2009) found that blueberries “Brigitta” treated with CA had a higher TA than control samples. Looking at ascorbic and citric acid concentrations, both were more stable in fruit subjected to GCA and CA berries than the control. This study showed the positive impact of GCA on preserving bioactive life of blueberry and potentially for other fresh produce by suppressing senescence decline. Both pH and TA remained stable across the experiment, while changes were observed in organic acid content. We acknowledged the lack of correlation among these parameters; this could be related to the lower sensitivity of pH and TA measurement methods, compared to HPLC.

The difference in the results between seasons 1 and 2 (*p* < 0.05) may be potentially attributable to variable environmental preharvest factors, particularly temperature as several heat waves were registered in June and July 2018 (Met Office, 2018).

## Conclusion

Blueberries require advanced postharvest techniques to maintain their physiological and functional quality. The development of an innovative CA approach, based on gradually reaching the optimal storage concentrations (GCA) allowed the reduction of blueberry respiratory metabolism when compared to standard CA and control treatments. This had a positive impact on quality parameters such as firmness and decay incidence. The reduction in respiration was also evident as sugars and organic acids were better maintained, these being the main respiratory substrates. GCA could potentially be successfully applied to other blueberry cultivars. Further research is needed to better understand the mechanisms underlying this technique and the efficacy of its use on other fresh produce.

## Data Availability Statement

All datasets generated for this study can be accessed at https://doi.org/10.17862/cranfield.rd.11876544.v1.

## Author Contributions

NF and LT conceived and designed the experiments. NF and TM performed the experiments. NF wrote the manuscript with the help of LT. All authors contributed to the discussion, revised, and approved the final manuscript.

## Conflict of Interest

The authors declare that the research was conducted in the absence of any commercial or financial relationships that could be construed as a potential conflict of interest.
