# Supplementary material for: Graduated Controlled Atmosphere: A Novel Approach to Increase “Duke” Blueberry Storage Life
Source: Front Plant Sci. 2020 Mar 17;11:221. doi: 10.3389/fpls.2020.00221 (PMC7092723; doi:10.3389/fpls.2020.00221)
Supplement: Supplementary file 1 [file Table_1.DOCX]

Graduated controlled atmosphere: a novel approach to increase “Duke” blueberry storage life

Natalia Falagán^1^, Tiana Miclo^1^, Leon A. Terry^1*^

^1^ Plant Science Laboratory, Cranfield University, Bedfordshire, UK, MK43 0AL, United Kingdom

*** Correspondence:**Prof. Leon A. Terry
l.a.terry@cranfield.ac.uk

Supplementary Material

# Supplementary Data

**Table 1** Total soluble solid content (TSS) expressed as %, titratable acidity (TA) expressed as g citric acid L^-1^ and pH of “Duke” blueberry subjected to controlled atmosphere (CA) applications throughout 28 days of storage at 0 ºC and 90 % RH; air [control]; standard CA (10 kPa CO_2_ + 5 kPa O_2_); graduated CA 7 (GCA7; 7 d to achieve standard CA values); and GCA3 (3 d to achieve standard CA values). Two consecutive seasons are considered 2017 (year 1; Y1) and 2018 (Y2). Data represents means (n = 10). Least Significant Differences (LSD) are shown.

| Time (days) | Treatment | pH | | TSS | | TA | |
| --- | --- | --- | --- | --- | --- | --- | --- |
|  |  | Y1 | Y2 | Y1 | Y2 | Y1 | Y2 |
| 0 | Baseline | 3.93 | 3.50 | 11.90 | 13.07 | 3.14 | 4.32 |
| 7 | Control | 3.67 | 3.53 | 11.10 | 12.73 | 2.66 | 4.89 |
|  | CA | 4.10 | 3.54 | 12.17 | 12.17 | 2.43 | 3.88 |
|  | GCA7 | 4.24 | 3.50 | 11.60 | 12.43 | 2.46 | 6.95 |
|  | GCA3 | 3.98 | 3.80 | 11.30 | 12.63 | 3.87 | 2.98 |
| 14 | Control | 4.12 | 3.66 | 12.83 | 13.50 | 2.64 | 2.81 |
|  | CA | 3.96 | 3.29 | 11.47 | 12.07 | 3.09 | 4.36 |
|  | GCA7 | 4.08 | 3.48 | 11.97 | 12.37 | 2.88 | 4.03 |
|  | GCA3 | 4.01 | 3.62 | 11.80 | 12.60 | 3.15 | 3.34 |
| 21 | Control | 4.04 | 3.24 | 12.27 | 12.90 | 3.23 | 5.50 |
|  | CA | 4.18 | 3.31 | 11.80 | 12.37 | 2.63 | 5.02 |
|  | GCA7 | 4.04 | 3.31 | 11.57 | 13.03 | 3.31 | 4.99 |
|  | GCA3 | 4.14 | 3.32 | 11.67 | 12.60 | 3.19 | 4.19 |
| 28 | Control | 3.77 | 3.43 | 11.90 | 11.67 | 3.38 | 4.41 |
|  | CA | 4.14 | 3.45 | 11.30 | 11.77 | 2.63 | 4.36 |
|  | GCA7 | 3.96 | 3.41 | 12.27 | 11.93 | 3.31 | 4.08 |
|  | GCA3 | 4.07 | 3.44 | 12.26 | 11.90 | 3.19 | 4.32 |
| LSD *– Time* | | NS | 0.19 | NS | NS | NS | NS |
| LSD *– Treatment* | | NS | NS | NS | NS | NS | NS |
| LSD *– Time * Treatment* | | 0.20 | NS | 0.32 | NS | NS | NS |

NS = non-significant
